# The unseen use of antimicrobials: Drivers of human antibiotic use in a community in Thailand and implications for surveillance

**DOI:** 10.1080/17441692.2023.2298940

**Published:** 2024-01-08

**Authors:** Nour Alhusein, Nutcha Charoenboon, Kantima Wichuwaranan, Kornrawan Poonsawad, Varapon Montrivade, Matthew B. Avison, Luechai Sringernyuang, Helen Lambert

**Affiliations:** a Population Health Sciences, Bristol Medical School, University of Bristol, Bristol, UK; b Faculty of Social Science and Humanities, Mahidol University, Nakhon Pathom, Thailand; c School of Cellular and Molecular Medicine, University of Bristol, Bristol, UK

**Keywords:** Antimicrobial use, Antimicrobial consumption, Thailand, Antimicrobial resistance, AMR national action plan, Antibiotic use

## Abstract

We investigated sociocultural and economic drivers of human antimicrobial use (AMU) in Thailand through ethnographic research, interviews, focus groups and a cross-sectional survey. This community-based study generated findings clustered around three key themes: treatment-seeking practices, medicine use, and interpretation of biomedical constructs. Participants sought care from public health facilities for chronic conditions, but medicines from the private sector were considered more powerful and were preferred for acute complaints. Many antibiotics were unrecognised as such by consumers due to the practice at private healthcare facilities of dispensing repackaged medicines without identifying labels. This unseen use of antibiotics is probably driven by economic drivers including market competition in the private sector, policy implementation drivers whereby rational drug use policies mainly target the public sector, behavioural drivers relating to treatment seeking-practices, and sociocultural drivers that influenced participants’ understanding of medical terms and concepts. Participants regarded antibiotics as reducing inflammation and were uncertain about the distinctions between anti-inflammatories, antibiotics, and pain relievers. Antimicrobial Resistance (AMR) was understood as a form of drug tolerance to be remedied by changing the medicine. Community surveys may not provide accurate estimates of AMU where people are unable to distinguish antibiotics reliably from other medicines.

## Introduction

Antimicrobial resistance (AMR) accounts for an estimated 700,000 deaths per year and by 2030 will represent up to US$ 3.4 trillion in GDP loss (World Health Organization [WHO], 2019b). In 2019, AMR was listed as one of the top 10 global public health threats facing humanity (WHO, 2019a). This step came four years after WHO published its Global Action Plan on AMR, which set out a road map for a coordinated global response to combat AMR while emphasising the need for context-specific national action plans (WHO, 2015a). Widespread antibiotic use in humans and animals is considered a major driver of AMR, especially antibiotic resistance (Bronzwaer et al., 2002; Goossens et al., 2005; Mulchandani et al., 2023; Van Boeckel et al., 2019). Surveillance systems to measure rates of antimicrobial and particularly antibiotic consumption and use are crucial in efforts to control AMR (WHO, 2021b). In 2020, WHO published the GLASS methodology for surveillance of national antimicrobial consumption (WHO, 2020). In WHO terminology, ‘antimicrobial Consumption’ (AMC) is a proxy estimate of antimicrobial use. It is calculated using aggregated data sources such as import or wholesale data, or aggregated health insurance data, but provides little information on actual usage patterns. It is often used by countries with limited resources in data collection. ‘Antimicrobial use’ (AMU), in turn, refers to estimates derived from patient-level data. AMU data are considered more reliable and informative indicators of actual use as they provide disaggregated information on patient characteristics and indications of use; however they are labour-intensive and expensive to collect (WHO, 2020). This paper utilises these terminological distinctions in presenting findings and the terms ‘antimicrobial +/− antibiotic’ are used to refer to the bacterial aspect of the broader world of ‘antimicrobials’.

In addition to AMC and AMU, WHO developed the AWaRe classification of antibiotics to gain a better understanding of which antibiotics groups are being consumed (WHO, 2021a). In this classification, antibiotics are classified into three groups; Access (includes antibiotics of lower resistance potential), Watch (includes antibiotics that are at relatively high risk of selection for bacterial resistance) and Reserve (the ‘last resort’ options when all alternative treatments have failed or are not suitable). These indicators were developed to help provide metrics and tools to monitor the effects of national stewardship policies and to define targets.

Early research in Thailand has estimated that there are about 88,000 drug-resistant infections a year, 38,000 deaths annually and an economic loss of almost 0.6 percent of gross domestic product (GDP) due to AMR (Pumart et al., 2012). Thailand established its national action plan on AMR in 2016 (National Action Plans and Monitoring and Evaluation, 2017). Its interim report (Thai working group on Health Policy and Systems Research on Antimicrobial Resistance [HPSR-AMR], 2021) reported a 5.6% decrease in human antimicrobial consumption between 2017 and 2019 (from 54.6 DID^1^ to 51.6 DID). However, this decrease occurred in the use of antibiotics on the Access list of the WHO’s AWaRe classification, while consumption of antibiotics on the Watch list has increased by 14.9%, and by 63.0% on the Reserve list (although the latter comprises less than 0.1 DID of total consumption).

Thailand’s surveillance system, like that of many other countries, uses pharmaceutical importers’ and manufacturers’ data to monitor antimicrobial consumption (AMC) at national level (HPSR-AMR, 2017). More detailed contextual information on antimicrobial use (AMU) at the patient level is missing, especially in the community, and thus interventions are harder to formulate. Accurate measurement of community AMU is also challenging because of over-the-counter use (purchasing without prescription) which cannot be captured through health systems data.

Our study was part of a wider project (Booton et al., 2021) to build a holistic picture of AMR drivers in Thailand from the One Health (human-animal-environment) perspective. In this component, we investigated sociocultural and economic factors contributing to human antibiotic use in the Nakhom Pathom provincial region of Thailand, with the aim of supporting the national AMR action plan in generating further evidence that can help to guide policy.

## Methods

### Study area

The study area consisted of a district in Nakhon Pathom province. It is a mixed rural and semi-urban setting with a growing number of factories having been built in the area in recent due to its relative proximity to the capital city Bangkok. There has been a gradual shift from traditional agricultural occupations (e.g. rice farming) to working in factories and other farming ventures (aquaculture, poultry and pig farming). Until the 1990s, travel was not easy, and locals had to take a boat to reach the health centre. Since paved roads were constructed, there has been an increase in the number of healthcare providers and changes in people’s use of health services. At the start of the research period, the district had 19 retail pharmacies, 16 private clinics and 18 primary healthcare centres as well as a community hospital. Additionally, some medicines are sold without prescription from grocery stores. The combination of different human/agricultural/industrial activities in the area has driven its importance ecologically in relation to AMR and thus as a study area of AMR and AMU drivers.

### Study design

This study used mixed methods consisting of ethnographic observation and interviews, focus group discussions and a cross-sectional survey. The mixed methods approach allows for methodological triangulation which helps to develop a comprehensive understanding of a phenomena and increase the validity of results (Patton, 1999) (Carter et al., 2014).

### Interviews and focus groups

#### Participants, recruitment and sampling

Data collection took place between June 2020 and December 2021. Initially researchers contacted local village health volunteers (Kauffman & Myers, 1997) and explained the study. Contact with households was then made by the village health volunteers who purposively selected households based on health status (including households known to have had contact with healthcare services due to ongoing health problems), geographical area (representation from both rural and semi-urban areas) and socio-economic diversity (variety of occupations and economic positions). Households who were happy to participate were introduced to the researchers. In total, 40 households were recruited in which three social science researchers (KW, KP, NC) conducted participant observation and completed interviews with family members. Participant observation took place during repeated field visits to each household by three researchers that included participating in and recording (through note-taking and photographs) various household activities including domestic tasks, food cultivation in home gardens, and livelihood generation, as well as household use of medicines, characteristics of the surrounding environment and proximity to health care facilities. Interviewees were in effect self-selected from among family members according to availability, but efforts were made to ensure a representative spread of occupations and household types across the overall sample. Other adult family members contributed to interviews if they were present. Only adults participated in the study, but some informants spoke of their experiences handling younger household members’ illnesses during interviews. The total number of household members was 216, among whom 60 (28%) were elders (60 years old and over), 114 (53%) were adults (19–59 years old) and 42 (19%) were 18 years old or younger. Households’ sizes ranged between 2 to 13 members with an average of 5 members. Occupation varied but were mostly related to traditional agriculture, factory manufacturing or food industry. Households’ demographics can be viewed in (Supplementary data: Table 1). Additionally, two focus groups were conducted by (LS, VM, KW, KP). Focus group participants were recruited from two geographical areas (rural vs semi-urban) and included a combination of village health volunteers and lay people. The participants’ age ranged between 30 and 60 years and occupations included government employment, running private businesses or farming (Supplementary data: Table 2: Focus groups’ demographics).

#### Data collection

To establish rapport, researchers conducted several visits to each household. All visits were made on an invitation/permission basis from the key informant of the household. On average, at least 5 visits were made to each household. Monthly visits were made to some households with which the research team had developed deeper relationships. Time per visit varied from the early (30–-60 min) to the later (1–3 h) period of fieldwork. In these relationship contexts, consent was obtained initially through verbal communication and subsequently through confirmatory action. The three main themes for investigation included living conditions (livelihoods, food and water sources), health and treatment-seeking (including medicine use). Researchers used both audio recorders and field notes to record these visits (Supplementary data: Interview topic guide). Thus, study findings comprised both direct quotes (digitally recorded conversations directly from the participant) and indirect accounts (where the researchers wrote narrative accounts of events and conversations based on field notes) (Hammersley & Atrkinson, 2019; Nayiga et al., 2022). These methods were suitable in areas where research activities were not familiar and participants did not always feel comfortable being recorded. Focus groups discussions included topics such as participants’ familiarity with available antibiotics, their common usages, where they were obtained and participants’ understanding of antimicrobial resistance (Supplementary data: Focus groups topic guide).

#### Data management and analysis

Interviews and focus groups were conducted in Thai. All recorded interviews and focus groups were transcribed *verbatim*. Combined with the field notes, the transcribed materials were summarised into anonymised case reports for each household, translated into English, coded using NVivo (version 12) and analysed thematically (Braun & Clarke, 2006). We first used open coding followed by axial coding, in which codes were integrated into categories and subcategories, from which themes were identified. Illustrative quotes (direct and indirect) are presented with descriptors including the Interviewee ID, their age, sex and occupation. For example: HH103(1) means: **H**ouse**H**old (from area: **1**, household number: **03**) (member of the household:**1**).

### Survey

A cross-sectional survey on medicine use in the 40 households was conducted in March 2021 by three social science researchers (KW, KP, NC) at the end of the fieldwork period with the assistance of 10 graduate anthropology students from Mahidol University who were recruited and trained to help with survey data collection. The survey questionnaire was developed by the Mahidol University and University of Bristol research teams to cover three topic main areas: information on illnesses episodes (participants were asked if they had any of these symptoms in the last three months: fever, cough, sore throat, nasal congestion, diarrhoea, vomiting, wound abscess, urinary tract infection symptoms or others), treatment-seeking actions (where participants sought healthcare for these illnesses: selfcare, grocery store, pharmacy, clinic, health centre or hospital), and antibiotics use (whether antibiotics had been used and if so, which ones) (Supplementary data: Medicine use survey form). The questionnaire was piloted with local community members who were not among the participating households. Informed consent was obtained from all participants and all interviews were conducted by trained members of the research team. Survey data were entered using Epicollect interface (version. 5, https://five.epicollect.net), uploaded into in Stata v16 [StataCorp, 2019] and analysed. Descriptive statistics were produced to describe the use of healthcare services (public vs private) and reported antibiotics.

### Ethics

Formal ethical approval was received from Mahidol University Social Science Institutional Review Board, Thailand and registered with University of Bristol, UK. Certificate of approval No: 2019/ 026.0702.

## Results

### Cross sectional survey

**Figure 1:**
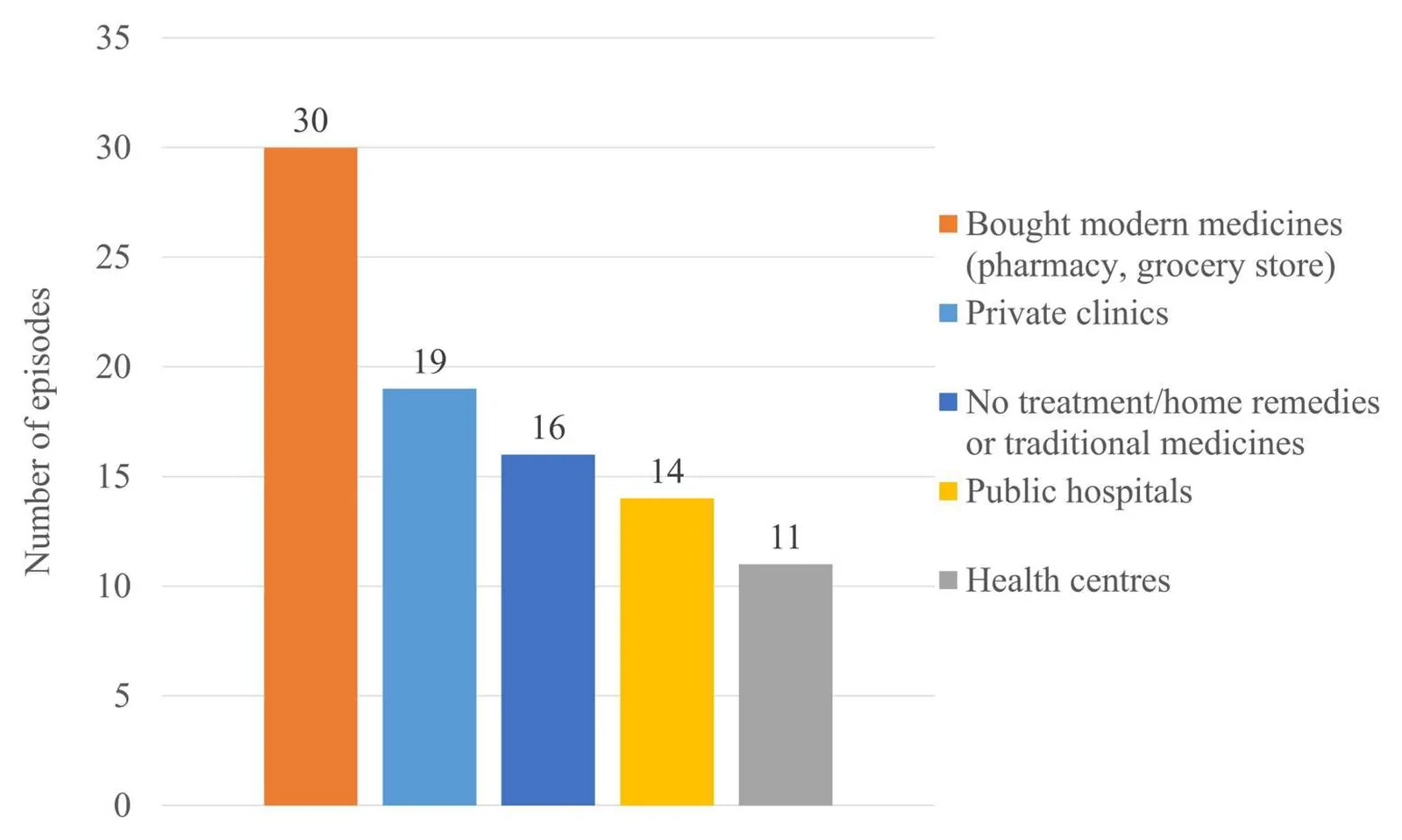
Treatment seeking actions of 88 disease episodes reported in the survey.

The survey documented 88 illness episodes among 188 individuals over three months. For these 88 illness episodes, 90 treatment seeking actions took place (In two cases participants reported more than one action: bought modern medicines from grocery stores/pharmacy, then went to a private clinic; and went to a private clinic, then bought medicines from a pharmacy/grocery store. Due to recall bias, the number of multiple actions may be higher than reported). Of those 90 actions, 49 (54%) sought care in the private sector (defined as including clinical facilities, pharmacies and over-the-counter purchasing of medicine from retail outlets), 25 (28%) in the public sector (including public hospitals and primary health centres) and 16 (18%) with home/traditional remedies or untreated (Figure 1). Only 15 treatment seeking actions (16.6%) overall reported using antibiotics including Dicloxacillin, Tetracycline, Amoxicillin, Cloxacillin, Norfloxacin and Roxithromycin. Antibiotics were sourced from pharmacies (n = 7), private clinics (n = 3) and health centres (n = 5). These antibiotics were used to treat diarrhoea (n = 1), upper respiratory tract infection (URTI) (n = 13), and wound infections (n = 1).

### Interviews and focus groups

**Figure 2:**
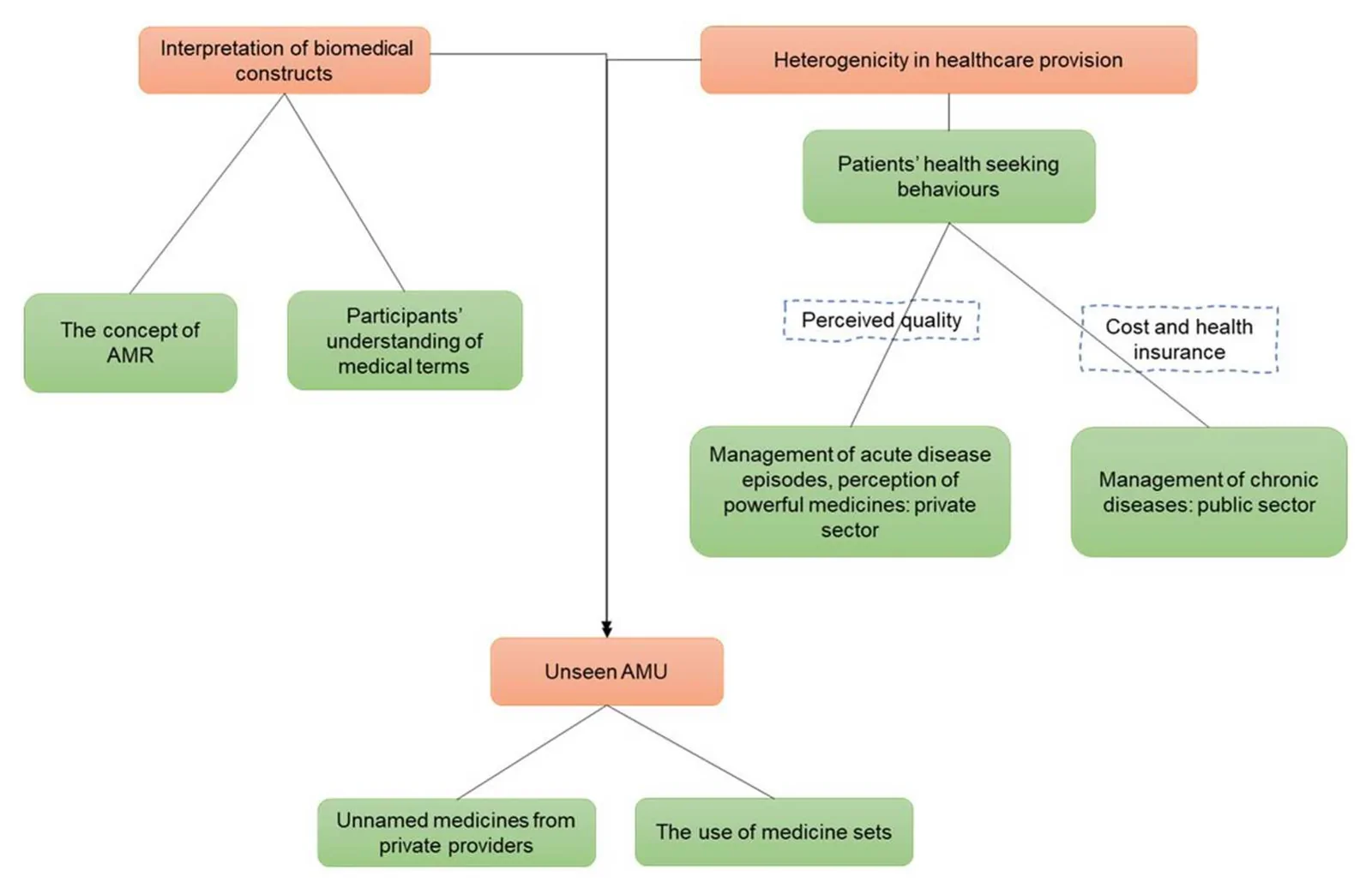
Identified themes and subthemes. Solid shapes: identified themes and subthemes, solid arrows: proposed relationships between the identified themes and sub-themes, dotted shapes: possible factors contributing to health seeking behaviours.

Three themes were identified (Figure 2): (1) Treatment-seeking in a heterogenous healthcare system, (2) The unseen use of medicines and antibiotics, and (3) Interpretation of biomedical constructs.

### Treatment-seeking in a heterogenous healthcare system

Numerous informants spoke about their experiences of treatment-seeking in relation to the variety of options available within the local health system and the constraints that influenced their decision-making about where to seek treatment. This heterogeneity formed a contextual background to the more specific themes described in subsequent sections.

### Heterogenicity of healthcare provision

Many participants reported variations in terms of access, quality, and financing between different healthcare providers. Under government universal coverage and the social security insurance schemes (WHO, 2015b), individuals are registered with a specific health care facility which is often close to their place of residence or work, but they may be referred to another more specialist public sector hospital if clinically required.

All of uncle HH118(1)’s children will have treatment at hospital (2) [central hospital]^2^ because they have their health insurance there, but uncle will have treatment at hospital (1) [community hospital] first because he has a gold card right [Universal Coverage Scheme]. Hospital (1) will decide whether send him to hospital (2) or not. (HH118(1), male, 60 years, farmer)

Participants described some public hospitals as having long queues, few resources and medical staff as being unfriendly and uncommunicative. The need for referral created access barriers to better resourced hospitals, as although self-referral was possible, this meant the cost not being covered by insurance.

He was prescribed some medication […] His health routine stayed this way for almost a year, but Uncle HH110(2) did not feel better. He started to have pain and his urine became milky white. Uncle believed this meant the ‘inflammation’ was getting worse […] Uncle said the doctor at Hospital (2) was impolite and did not write him a medical referral, so he decided to go to Hospital (3) himself as suggested by his acquaintance. He went to Hospital (3) by himself, without any referral, so he was not granted the Universal Health Coverage Scheme or the Senior medical right because the hospital was in another district. Consequently, he had to pay for the room, which was 700 baht^3^ per night, the daily antibiotic injections, which were 1,200 baht per injection, the daily meals, 150 baht, and other medication and medical service bills. (HH110(2), male, 68 years, braids baskets)

Private sector facilities were generally seen to provide a quicker and higher quality service that was locally accessible, and medical staff were described as more patient-friendly. However, the need to pay out-of-pocket for private care meant that although some participants could afford an occasional visit to a private clinic or to purchase medicines over the counter, longer term healthcare management through private clinics was recognised as costly.

They mostly go to the clinic because it is more convenient. The regular clinics are the ones in [area name] or [area name]. They said Hospital (1) [community hospital] does not provide good treatment and is too far from their house. (HH235(1) and (2), female (64 years) and male (71 years), farmers and own a grocery store)

Each visit to the clinic costs around 200–300 Baht. (HH225 (1), female, 56 years, cook)

In cases of chronic conditions, participants mostly used public hospitals due to the operation of the non-communicable diseases surveillance scheme, the high cost of managing ongoing conditions privately, and the ability to arrange referrals to more specialised facilities.

Even uncle HH118(01) had a check-up at the [private] clinic, he went to get the medicine from the [government] hospital. Because clinic costs a lot of money around 1,000 Baht, and he has to go get the medicine every 2 months. (HH118(01), 60 years, farmer)

When the result came out, the doctor [at the clinic] suggested going to Hospital (1) [community hospital] because he believed that the stomach aches was not originated from her gastritis. HH237(01), then, had an ultrasound scan at Hospital (12) and discovered that she had gallstones. (HH237(01), female, 55 years, opened a noodle and cook-to-order shop)

### Seeking powerful medicines

A key attraction of private providers was that they are perceived as more likely to provide medicines that work. Participants described such medicines variously as ‘strong’, ‘powerful’, ‘effective’, ‘eliminate the sickness faster’ or ‘reach the problem’, while referring to medicines from the public sector as ‘weak’, ‘basic’, ‘isn’t powerful’ or ‘don’t improve the condition’.

This clinic dispenses medicines at high cost, but their medicines work; ‘reaches’ the problem. Working means, it’s not that I imagine it myself, but they really help me feel better after I take them. (HH116(1), male, 37 years, fisherman)

Focus group participants who were village health volunteers commented that people tended to develop their own judgements on what worked based on how quickly their symptoms improved.

Participant C: When they take medicine provided by the health centre, they will be like, ‘Nah, this medicine is nothing. I’d rather go buy one myself’. Look, when someone gets sick, they go straight to the drug store without having a glimpse on the medicine from the health centre because it isn’t powerful. Participant B: And when you go to the health centre, they only give you basic medicine which takes 10 days or 3 days to be effective. People aren’t satisfied; they prefer those that only need 2 days so they can go back to work. Meanwhile, the medicine you got from the health centre needs many days. (Focus group 2)

### The unseen use of antibiotics

#### Self-medication with antibiotics

The most popular antibiotics among our research participants were TC Mycin [Tetracycline], Aureomycin [Chlortetracycline], Ha-Saen [Penicillin], Amoxicillin, Dam-Daeng [Dam (black) – Daeng (red): Tetracycline], and Gano [Tetracycline]. These medicines were used mostly to treat symptoms of cold and flu, sore throat and wound infections and participants would request them by name from a pharmacy or grocery store.

About the use of inflammatory, HH226(4) will take TC Mycin [Tetracycline], Aureomycin [Chlortetracycline], and Ha-Saen [Penicillin] for wounds. She says she only takes the medicine when the wounds are inflamed. The difference between normal wounds and inflamed wounds is the size of the wound. Big wounds, wounds that spread, and wounds that are reddened and swollen are inflamed. (HH226(4), female, 54 years, Makes artificial marigold garlands)

Participant 3: We have to see what is occurring first. For wounds and sore throat we use Amoxicillin. If it is inflammation, Penicillin or TC Mycin [Tetracycline] are the ones to go. (Focus group 1)

Other antibiotics were rarely recognised by name, meaning that participants would not be able to obtain them without help from a provider. For example, one participant explained that she often asked the pharmacist to give her a “good anti-inflammatory (‘Ya-Kae-Àk-sàyp’) medication”. ‘Ya-Kae-Àk-sàyp’ means ‘anti-inflammation medicine’ and is also the most commonly used term for antibiotics. The provider in this situation often decides what to dispense based on the presentation. If a client comes with sore throat or cold, ‘Ya-Kae-Àk-sàyp’ would be taken to mean antibiotics. If a client presents with muscle pain this request may lead to the provision of anti-inflammatory drugs.

HH120(01) also has anti-inflammatory drug at home. Aunt understands that this drug can prevent swelling or inflammation. Everyone in the house takes this type of medicine especially when there is an accident, for example, a spine of a fish pricks a finger. The anti-inflammatory drug is a green and black pill. Aunt told the researchers that she must tell the pharmacist that she needs a good anti-inflammatory (‘Ya-Kae-Àk-sàyp’) medication and she will be prescribed with the green and black pill at the price of 50 baht per packet. Nevertheless, she sometimes purchases anti-inflammatory pill from the general store near her house at the price of seven baht per pill. (HH120(01), female, 65 years, farmer)

Many participants reported obtaining medicines without knowing exactly what they were, meaning that there was likely to have been a degree of under-reporting in our survey data.

Participant D: It’s [referring to a capsule] for sore throat, coughing, cold. Interviewer: So, you are familiar with this because it comes with the medicine for cold and sore throat. Participant B: Yes. Participant C: Sore throat. Interviewer: To be clear … you don’t know what’s its name, right. Participant B: I don’t know because it comes in pack. (Focus group 2)

We characterise this as ‘unseen’ use of antibiotics and it took place through two routes, as described in the following sections.

#### Unnamed medicines from private providers

**Figure 3:**
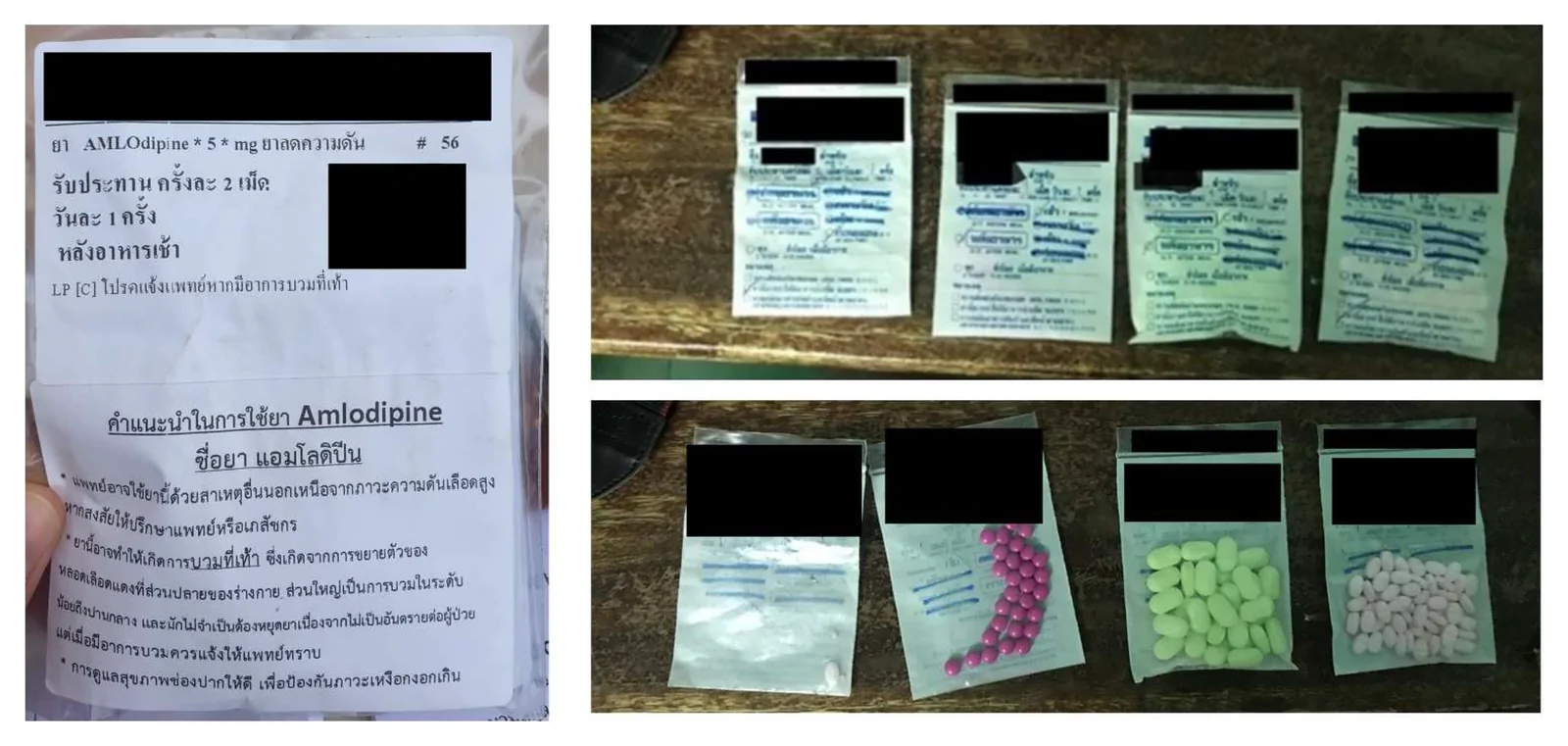
Right hand (top and bottom) images: Medicine packs dispensed from private clinics or pharmacies. The label gives the clinic logo (anonymised), patient name (anonymised), administration instructions and contraindications, but not the name of the medicine. Left hand image: a medicine pack dispensed from a public hospital (name redacted). Label includes medicine name, instructions for use, patient information note.

In both government and private clinics, pharmacies and hospitals in Thailand, prescribed medicines are frequently dispensed to patients in labelled plastic bags (each containing one type of medicine) rather than their original packaging. As verified by direct observation (Figure 3), however, repackaged medicines dispensed from private (not government) health care facilities were labelled with dosage and other details but frequently lacked the name of the specific medication. In some cases, participants noted that private healthcare providers deliberately tore the labels off medicines before dispensing them. According to some participants, this was to prevent patients from subsequently obtaining the same treatment from another provider.

The private hospital won’t let us see the prescription. Participant B: The private hospitals won’t tell us anything. Participant A: They just give us the medicine right away. Participant B: It’s all pre-packed ready to retrieve. You pay and take it. Participant C: They also tear off the label attached on the syrup bottles. Participant B: They tear it off. Participant C: They tear off the name of the medicine, only information about the dose is left. Interviewer: Why do they do that? Participant B: To keep it from us. Participant C: They might not want us to know. Participant B: If we know, we’ll go buy the medicine ourselves. For example, if the anti-inflammatory is the disinfectant dissolved in water, they keep it from us, because if we know we can go to the drug store and tell them what we want. (Focus group 2)

The doctor gives me 15 pills of an unnamed medication. It comes in a set with the clinic’s name on the bag. I don’t take all the 15 pills. Only take it when the skin is itchy. I went to pharmacy [pharmacy name] and asked for it but they didn’t have one as the pharmacist said it was imported. (HH112(01), male, 58 years, village head, health volunteer, produces and sells products from his garden)

Participants believed that medicine packs were individually tailored for them and held that once they were attached to a specific private provider, they could not go elsewhere due to the provider not disclosing details of their prescription.

Participant E: Changing the clinic isn’t good. I’m not sure. It might be because of the different medicine. Let’s say, the medicine from one clinic might not be as effective as another’s. If the first clinic gave you a very strong medicine, the weaker medicine from another clinic won’t be effective because your body’s familiar to the strong substances. Participant E: You get addicted once you take it, you’ll have to keep buying from the same place because the medicine from other stores won’t help. Participant B: It must be this store. Only medicine from this store could help, the other stores’ medicine couldn’t help. Participant E: No, they give different medicine although it’s the same symptom. Participant C: Sometimes we notice that the medicine’s different from what we usually take, so we have to go back to the same store. (Focus group 2)

Participants also spoke of receiving injectable medicines from private clinics without identifying the injectables they received and referred to them descriptively (e.g. ‘getting an injection’, ‘cough injection’, ‘a flu injection’). Participants mentioned that injections in particular were known to be more accessible from private clinics and were considered a ‘stronger’ form of treatment for use in what the participants perceived as more serious cases of illness.

HH109(01) has heard that people have to go to clinics to get an injection because each time it costs around 500-600 baht, that it is easier to get injections at clinics than hospitals, and that injected medicines are stronger than medicines taken orally making the patient return regularly for the same injections. (HH109(1), female, 70 years, braids baskets)

The family prefers going to a clinic over the hospital when they are sick. Doctor [doctor name] and doctor [doctor name] clinics are regular places they visit because they provide a jab [an injection] that works better than the medicine from the health centre. (HH223(01), female, 59 years, housewife, casual breeder of chicks)

She had been visiting [doctor name] clinic and treated by an injection and body cream to be applied at home. She had no idea what the cream was for. (HH111(1), female, 57 years, braids baskets)

Some participants mentioned that they specifically went to a certain clinic because they felt that receiving an injection from that clinic would cure their illness effectively; and that once one is used to injectable medicines, oral medications no longer work.

The hospitals around here don’t help [improve the symptoms], but when we went to doctor [doctor name], he did. So, from then, whenever someone is sick, we would only go to doctor [doctor name]. When we go, we’d receive medicines and an injection, and we recover. He judges [the treatment] by the symptoms. If it’s a lot and seems like it won’t get better, he would give an injection. But if it’s not a serious illness, he would give us medicines and it’ll be better, recover. (HH103(3), female, 58 years, cook)

But if you get an injection, you would get used to them, you wouldn’t really get better if you take pills, you would then need injections more regularly and frequently, and the doctors at the clinic and at the hospital are the same doctors, but it feels like the clinic’s medicines help eliminate the sickness faster. For example, if you have a fever 3 times this year and you go get 3 injections, the pills won’t help anymore so you have to go back to get more injections. And the injections are stronger, but the pills are weak. (HH109(1), female, 70 years, braids baskets)

#### The use of medicine sets

**Figure 4:**
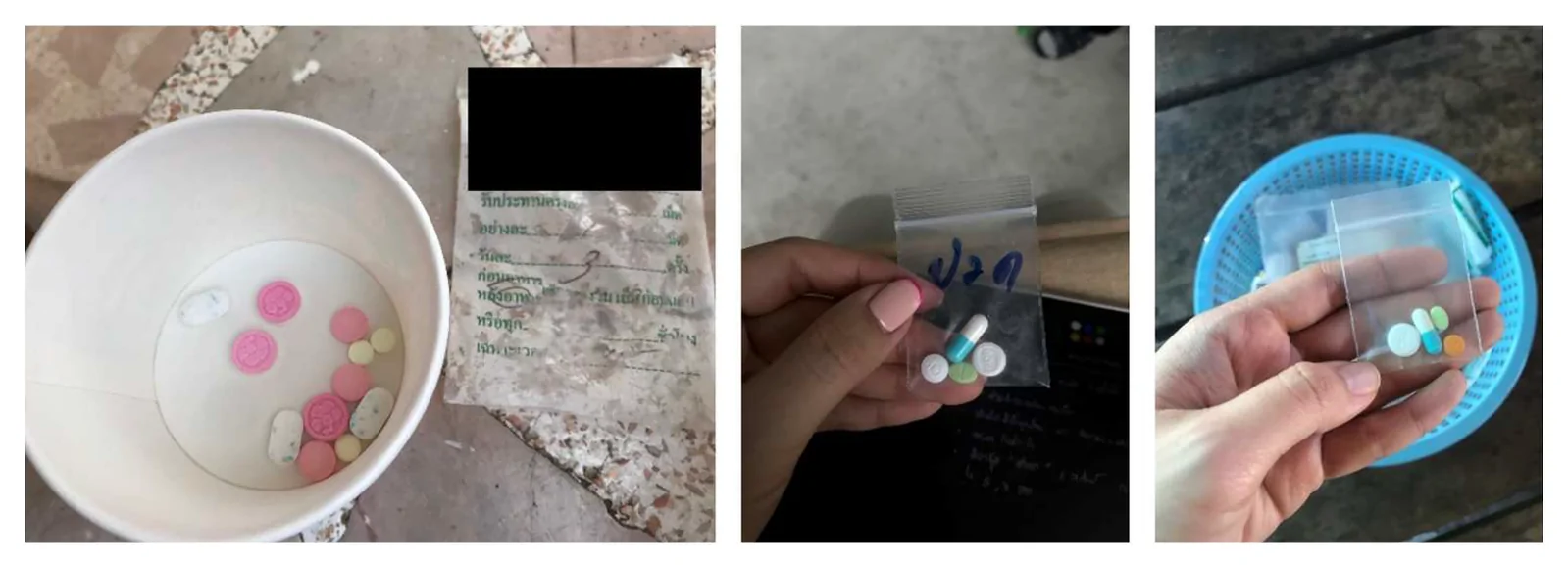
Medicine sets (*Ya Chud*) packed in plastic bags, left hand image: writing on the bag specifies frequency of administration. Middle image: writing on the bag states: ‘for pain’. Right hand image: Unlabelled.

The term ‘Ya Chud’ (literally, ‘medicine set’) refers to a mixed set of medicines packed in a transparent plastic bag with few instructions (Figure 4). They have long been popular in Thailand (Sringernyuang, 2000), and despite being officially prohibited from sale and heavily stigmatised among healthcare professionals, we found that they remain available in some grocery stores and some pharmacies. What each medicine set contains is difficult to determine as they are labelled according to the symptom or complaint they are intended to treat (e.g. pain, cold) and the information on the packaging does not include the medicines’ names (Sri-Ngernyuang, 1996). Some participants who were health volunteers explained that people tend to request ‘Ya Chud’ for cold and flu treatment, rather than asking for specific medicines. Accordingly, customers who purchase medicine sets often have little knowledge of what these bags contain.

Drug cocktails [ยาชุด – ya chud] mean when you go buy them from shops, it can be this drug store here. You don’t have to go far, just that drug store where … Say, I have a cold. Instead of, you see [hands interviewers a decongestant] and one pill would already do, they would mix that and this together. A pharmacy would do that. But we can’t know at all what medicines those are. Say, if I go and tell them I want one pack of [decongestant commercial name] and one pack of [cold and flu medicine commercial name] because I know what [disease] I have – namely a cold – then that’d be it. But if I told them, ‘Arrange a set of cold medicine cocktail for me’, then they would put it together for me, the pharmacist would. The villagers wouldn’t know, they wouldn’t. Some people would go, ‘Doctor, I have a cold. Arrange a set of medicine for me’. Then the doctor would put together a set(s) for them. Whatever the doctor prescribes, they would take it. (HH234(1), female, 52 years, farmer)

Aunt HH229(1) had bought some medicine herself, which it was mostly ‘flu medicine’ or ‘Ya-Chud’, a variety kind of medicine all mixed into a small bag with each ‘Chud’ or bag consists of 3-4 medicines. She would buy a Chud that consists of anti-inflammatory drugs, cough medicines, and aspirin from [name] Pharmacy in [town] Market. She said that [person’s name] was once working as a pharmacist, then he opens his own pharmacy. When people get sick, they can simply tell him their symptoms, then he would prescribe the Chud accordingly. (HH229(1), female, 57 years, pig farmer)

Some participants preferred to take medicines sets as they perceived them to be more effective than the medicines prescribed at the (government) hospital. One participant described stockpiling medicines sets for flu and for pain and sharing them with her family and employees.

She says the medicine set [for pain] is more effective than the medicines she got from the hospital as it takes effect more quickly though she does not always take them. The hospital only gives paracetamol and relaxant. (HH223(01), female, 59 years, housewife, causal breeder of chicks)

This is a muscle relaxant. And this is for flu. I buy around 10 sets of them each time. When my employees come to help with [work] they can take them too. I take some muscle relaxant when my back gets hurt. I will take five pills of these if I have flu … I only take the medicine when I have the symptom. If the pain is severe, I will take three sets of the medicine after my meals, one per meal, consecutively. They only say to take them after the meal. Some says 20 min, and some says 15 min after. My employees take them. Sometimes, my son-in-law also takes one or two sets when his back hurts. (HH120(01), female, 65 years, farmer)

### Interpretation of biomedical constructs

#### Participants’ understanding of medical terms

Participants expressed uncertainty over the distinctions between anti-inflammatories, antibiotics, and sometimes even pain relievers. Many participants referred to antibiotics as anti-inflammatories used to treat inflammation symptoms of the upper respiratory tract (e.g. sore throat).

Whenever I get sick or have a sore throat, I would take the blue and green capsules. It is both an anti-inflammatory and antibiotics, a two-in-one … it does everything. (HH229(1), female, 57 years, pig farmer)

Interviewer: What about anti-inflammation and antibiotics? Can people differentiate the two? Do they know how different both are? Participant 1: Some know, some do not. Mostly, do not. Participant 2: Is antibiotic paracetamol? Participant 3: It is germ killing medicines. (Focus group 1)

Some participants understood anti-inflammatories (referring to antibiotics) as antiseptics used to clean wounds and prevent ‘germs’. Other participants used a term, that literally translates as ‘germ-killing medicine’ to refer to antibiotics used to treat diarrhoea.

On antibiotic understanding, HH102(1) told the writer that he knew about it from the advertisement rather than from the doctors, but he does not know what it is used for. When asked about anti-inflammatory, they believed that it is for cleaning wounds and preventing germs. Betadine and rubbing alcohol are some of them. (HH102(1), male, 47 years, owns a shrimp farm and leases a land)

Participant B: Just like what I said, germ killing medicine is the medicine for kids. Interviewer: Do adults take the germ-killing medicine? Participant B: Adults also take it when we have diarrhoea. Participant D: You take the germ-killing medicines when you have diarrhoea; that’s all. (Focus group 2)

Similarly, many participants referred to anti-inflammatories (non-steroidal anti-inflammatory drugs) as muscle relaxants or ‘tendon relievers’ used to treat the inflammation (e.g. swallowing, pain) of the relevant body part. These medicines were often known to be acidic to the stomach as a side effect. Terminologies were often associated with what symptoms patients experienced and medicines were referred to accordingly (e.g. ‘a fever medicine’, ‘pain medicines’, ‘muscle relaxant’).

Interviewer: You know there’re some kind of medicine, like, when you have a muscle inflammation, the doctor gives you the anti-inflammatory. Participant C: Yes. Participant B: Muscle reliever. Interviewer: Is that an anti-inflammatory? Participant B: I know it works for inflammation, but I don’t know what it is. When you go to the doctor, you get a muscle reliever. It’s acidic to stomach. It pierces through your stomach. Participant C: Sometimes it’s tendon reliever. (Focus group 2)

#### The concept of AMR

Antimicrobial resistance was understood as a form of ‘drug tolerance’, in the sense that the drug is considered to stop being effective due to overconsumption or frequent use. Additionally, participants explained that consuming ‘stronger’ medicines meant that the body becomes accustomed to them, so that the same strength or stronger medicines must be used to obtain a sufficient effect:

Let’s say, the medicine from one clinic might not be as effective as another’s. If the first clinic gave you a very strong medicine, the weaker medicine from another clinic won’t be effective because your body’s familiar to the strong substances. (Focus group 2)

These concepts did not apply solely to anti-inflammatories or antibiotics, or indeed to oral modes of administration alone:

But if you get an injection, you would get used to them, you wouldn’t really get better if you take pills, you would then need injections more regularly and frequently. (HH109(1), female, 70 years, braids baskets)

Accordingly, participants suggested that the solution to ‘drug resistance’ was to ‘change the medicine’.

I’ve heard about drug resistance. It’s when you take too much medicine. Look at paracetamol. We take it to ease a headache. Once you take it over and over, it can’t cure the pain anymore. This’s drug resistance. Then we need to change the medicine. (HH104(01), female, 72 years, braids baskets and grows vegetables)

Interviewer: Now, this’s the last question. What’s the solution for drug-resistance? […] Participant B: Change the medicine. Participant C: Change the medicine. Participant E: Mostly, we use another medicine. Participant B: Once you change the medicine, the problem’s solved. Participant C: Just change. (Focus group 2)

## Discussion

This study aimed to identify the drivers for AMU in a community setting in Thailand. Our findings indicate that antibiotics originating in the private sector (both retail outlets and clinical facilities) are not necessarily recognised as such by those consuming them due to medicines being repackaged and dispensed without identifying labels. Participants considered medicines from the private sector to be powerful, leading many to prefer private care (if they could afford it) for acute complaints. There was uncertainty around the interpretation of different medical constructs such as anti-inflammatories vs antibiotics and the concept of AMR. These identified factors contribute to continuing AMU through wider interwoven economic, policy-related, behavioural and sociocultural drivers.

Our findings indicate that the use of antibiotics is characterised by incomplete labelling of dispensed medicines as well as reported frequent use of unspecified injectable medicines in the private sector, as well as the use of medicine sets. Several economic, behavioural, cultural and policy implementation factors – market forces of competition over consumers in the private sector, consumers’ demand for injectables for acute illness, and limited adoption of rational drug use policies beyond the public sector – drive this unseen use of antibiotics. Historically, injectable antibiotics and other medicines were at one time widely available in rural Thailand through unqualified ‘injection doctors’ (Cunningham, 1970); in our study we found that our participants continue to prefer injections over oral medication as treatment for illnesses perceived to be serious. These injections were easily accessible from private clinics, though participants could not specify their contents. Medicine sets, or ‘Ya Chud’, which remain popular among sections of the rural population and can be purchased freely from some grocery stores, also contribute to the volume of unseen medicines. Several studies have found that ‘Ya Chud’ for cold and flu is likely to contain antibiotics (Sri-Ngernyuang, 1996; Kamolratanakul et al., 1992; Maluangnon et al., 2014; as cited in Sunpuwan et al., 2019). In Thailand, antimicrobial stewardship or ‘rational use’ initiatives to date have largely been implemented in the public sector (Pumtong et al., 2020). For example, the Antibiotic Smart Use project (ASU) (WHO, 2012), (Sumpradit et al., 2012) was a research/community initiative which was later adapted by the Ministry of Public Health and implemented in public hospitals (as Rational Drug Use Service Plan) (Waleekhachonloet et al., 2021). Uptake in the private sector is always more difficult, in part because variations in healthcare financing systems may influence the uptake of antibiotic stewardship practices. For providers that operate on a capitation payment system^4^, rational AMU policies are incentivised because they lead to lower expenditure per patient. However, for providers that operate on a fee-for-service scheme, as occurs in the private sector, implementation of antimicrobial stewardship could result in a reduction of income from antibiotic prescriptions (Sumpradit et al., 2012). The issue of balancing profit incentives and professional practice in pharmacies in low – and middle-income Asian settings has been described previously (Miller & Goodman, 2016). Their study indicated that although improving providers’ knowledge was necessary, it wasn’t sufficient to ensure good practice and that several ongoing profit-maximising strategies were leading to poor practice. Understanding antibiotic dispensing from this perspective invites a view of ‘inappropriate’ antibiotic use not just as a knowledge deficit problem of end users but as a product of economic and political conditions that requires interventions targeting societal structures (Tompson & Chandler, 2021; Charoenboon et al., 2019).

We characterise the unrecognised use of antibiotics as ‘unseen’. This is intended to highlight that this problem does not solely exist at consumer level, where we have shown that patients sometimes consume drugs that they do not know to be antibiotics, but also captures the additional implication that these antibiotics remain ‘unseen’ by both surveillance systems and policy actors. Methods of measuring antibiotic use based on self-report, such as community surveys and exit interviews, are likely to systematically underestimate antibiotic consumption when these medicines are not labelled or recognised as such, as documented in work in China where around half of patients attending both clinical facilities and retail pharmacies could not confirm whether their prescription or purchase contained antibiotics (Lambert et al., 2023). These absences in turn mean that this antibiotic use remains ‘unseen’ and hence unaddressed in policy. There is a need for national action plans to consider other sources of antibiotics within communities that remain ‘unseen’ by stewardship campaigns. Even in countries where antibiotics are only available on prescription, the scale of self-medication with leftover antibiotics is less explored and often remains unrecognised by policy makers (Machowska & Stålsby Lundborg, 2018; McNulty et al., 2006).

Our study also provides further evidence of the widespread use of antibiotics both with and without prescription in a rural community. Several regulatory and behavioural drivers play into (or against) this use. The 1987 Thai Drug act classified most antibiotics as ‘dangerous drugs’ that should only be dispensed by a licenced pharmacist, but only a few antibiotics are classed as prescriptiononly (Sommanustweechai et al., 2018). In 2019, as part of ongoing efforts to reclassify antibiotics as prescription-only (Sumpradit et al., 2021) in order to reduce easy access and self-medication practices among the public, all injectable antibiotics became prescription only. Yet successes in implementing rational AMU policies in government healthcare facilities may inadvertently have reinforced public perceptions of medicines from the public sector as ‘weak’ and contribute to treatment-seeking from private providers, particularly for accessing injectable medicines, including antibiotics. Antibiotics can be seen as a ‘quick fix’ used to correct fractured infrastructures of care, hygiene, water and increased demand for productivity (Denyer Willis & Chandler, 2019). In our study, the perceived need for stronger medicines or medicines that work was often associated with participants’ understandings of what good care means, and with the relentless requirement to be economically active. This resonates with what has been observed elsewhere in settings with minimum resources, in which care is characterised by provision of medicines, and restrictions on dispensing medicines to patients conflict with the concept of good care (Nayiga et al., 2022).

Participants in our study area were only able to name a few antibiotics, all of which are on the WHO Access list (WHO, 2021a). This indicates that the use of antibiotics on the WHO Watch list probably occurs only at the instigation of a healthcare provider. Reports on the extent of self-medication in Thailand are mixed. In a 2017 national household survey (Chanvatik et al., 2019), the prevalence of antibiotic use for three common conditions; flu, fever and sore throat among 27,762 adults in the preceding month was 7.9%. The prevalence of antibiotics purchased without prescription in a sample of 2024 adults was 29.7%. In another study comparing communitybased antibiotic access and use practices across six communities in low- and middle-income countries, the proportion of households in Thailand reporting use of antibiotics in the previous month was 27.9% (294 out of 1053 households). The proportion of antibiotics dispensed without prescription was estimated as 3.9% (18 out of 462 exit interviews with customers) (Do et al., 2021). The same study identified that in the Asian communities studied, over 90% of mapped antibiotics suppliers were private providers (95% (278/293) in Thailand) probably due to a high density of small private clinics and pharmacies, providing accessibility and convenience. Self-medication trends are often influenced by past successful use (Radyowijati & Haak, 2003). The AMU practices of prescribers, dispensers and patients in any given setting are often closely aligned and mutually reinforcing as they share similar cultural perceptions of illness and health (Radyowijati & Haak, 2003). Thus, the influence of clinical professionals’ prescribing practices on self-medication and dispensing practices needs to be considered when designing interventions to address over-the-counter dispensing and use of antibiotics.

Semantic differences between biomedical and local languages of health also contribute to the continuing use of antibiotics in the community. Participants in this study commonly associated ‘antibiotics’ with ‘anti-inflammatories’ and sometimes with other medicines for pain relief. Ambiguities in translation of biomedical terms into local vocabulary also contributes indirectly to clinically unwarranted AMU. The standard biomedical translation for ‘antibiotic’ in Thai is *Ya-Pa-Ti-Che-Wa-Na* or *Ya-Taan-Chun-La-Cheep*, both literally meaning ‘medicine against the tiny living’ (Sringernyuang, 2000), (Sri-Ngernyuang, 1996), (Haenssgen et al., 2019), (Chuengsatiansup et al., 2000). Another term, *Ya-Ka-Chue* (anti-infective agent), is also frequently used by medical professionals. However, in the public domain, *Ya-Kae-Àk-sàyp*, literally ‘medicine to cure Àk-sàyp’, is mostly used to refer to antibiotics. ‘*Àk-sàyp*’ in turn is rooted in Thai traditional medicine and means ‘inflammation’ (often indicated by symptoms such as abscess, swelling, redness, heat, and pus). However, health professionals also often use the term *Àk-sàyp* as a suffix to denote conditions affecting a specific organ or body part. Thus, conjunctivitis is *Ta-Àk-sàyp* (eye inflammation), while appendicitis is *Sai-Ting-Àk-sàyp* (appendix inflammation), muscle strain is *Klam-Nu e-Àk-sàyp* and pneumonia, *Port-Àk-sàyp*. These terms reinforce public perception of antibiotics (*Ya-Kae-Àk-sàyp*, literally ‘medicine to cure inflammation’) as appropriate treatment for a wide range of conditions. This creates a blurred and confusing conceptual link between antibiotics and inflammation similar to that found elsewhere in Asia (Lambert et al., 2019), (Wang et al., 2019). Societal knowledge building around antimicrobial resistance and the appropriate use of antibiotics is crucial to address this mismatch between biomedical and local understandings of inflammation, which is also found elsewhere in Asia (Lambert et al., 2019). The 2017 national household survey (Chanvatik et al., 2019) found that only 42.9% of respondents correctly responded to the statement that antibiotics are not anti-inflammatory drugs and 52.3% considered the statement that antibiotics can cure common cold and flu symptoms to be correct. In 2020, as part of the Thailand’s national strategic plan on AMR, a series of social marketing health campaigns targeted 9.8 million people within 1.5 months, and an awareness campaign led by the village health volunteers network, was launched to spread key messages on AMR and appropriate use of antibiotics. The campaign recognised the need to incorporate other governmental agencies (e.g. education ministry) and nongovernmental organisation (civil society organisations) to tackle the issues related to AMR and antibiotics awareness (Sumpradit et al., 2021).

The study has several limitations. It only captures the perspective of service users (the perspective of service providers is ongoing in our analysis). Most of our key informants were older people due to them being more available to participate in the study. The survey was conducted at the end of the field work due to earlier pandemic restrictions, by which time participating households may have been sensitised to the questions around antibiotics. However, the findings across both quantitative and qualitative methods were consistent, providing method triangulation and increased validity. The findings are specific to the research area and may not be generalisable to the entire population, although our findings are broadly consistent with and add to existing literature.

## Conclusions

The dispensing and purchase of unlabelled medicines in the private sector in Nakhom Pathom in Thailand means that patients are not necessarily aware they are receiving antibiotics and therefore methods of measuring antimicrobial use based on self-report (exit interviews with patients, community surveys) may not provide accurate estimates. To our knowledge this is the first study to identify specific practices in Thailand’s health sector that limit patients’ ability to identify what medicines they are using, and the first to highlight the implications for accurate surveillance of AMU. Further attention needs to be given to the development of innovative methodologies to track antimicrobial use at community level and to antimicrobial stewardship interventions that take into consideration shifts in meaning when biomedical terms are translated into local terminologies.

Our findings indicate that efforts are needed to extend antibiotic stewardship initiatives as part of rational drug use policies into private healthcare. Additionally, our findings suggest that the laudable initiatives to limit antibiotic prescribing in government health facilities may have unforeseen effects on treatment-seeking practices in the community. Future interventions need to bear in mind public responses to changes in drug use policies and to their implications for access to appropriate treatment.

## Supplementary Material

Supplemental data for this article can be accessed online at https://doi.org/10.1080/17441692.2023.2298940.

Supplemental material

## Notes

1 DID is defined as Defined Daily Doses (DDD)/1000 inhabitants/day.

2 Community hospitals provide primary care. General hospitals provide secondary care. Central hospitals provide tertiary care. At the top level of the system, there are medical school hospitals. Source: World Health Organization. WHO Regional Office for the Western Pacific. (2015b). *The Kingdom of Thailand health system review* (ISBN: 9789290617136).

3 Average exchange rate in 2021: 1 baht = 0.0313 USD. https://www.exchangerates.org.uk/THB-USD-spotexchange-rates-history-2021.html#:~:text=Average%20exchange%20rate%20in%202021,USD%20on%2005%20Dec%202021. [accessed 12/07/2023].

4 ‘capitated payment, or capitation, means paying a provider or group of providers to cover the majority (or all) of the care provided to a specified population across different care settings. The regular payments are calculated as a lump sum per patient’ from: Capitation: an introduction. UK government website. https://www.gov.uk/guidance/capitation (accessed 12/07/2023).
