# Supplementary material for: The unseen use of antimicrobials: Drivers of human antibiotic use in a community in Thailand and implications for surveillance
Source: Glob Public Health. Author manuscript; Available in PMC 2026 Sep 7. (PMC7619281; doi:10.1080/17441692.2023.2298940)
Supplement: Supplementary file 1 [file EMS215594-supplement-Supplemental_material.zip › Focus_groups_topic_guide.docx]

Focus groups topic guide

1. Introduction: Introduce facilitator’s background, objectives, key questions and the process of the interview.
2. Contextual Information: Historical, socio-economic accounts of the community? Socio-economic background of participants: details of age, occupation, family structure and relationship, and daily life
3. Health conditions
   3.1. General health problems- what are the common illnesses experienced by the participants?
   3.2. What kinds of health service facilities are available in the area? How do participants do when they get sick?
   3.3. Where do they commonly get treatment and medicines? Where do they usually get drug for self-medication?
   3.4. Do participants know or have ever experienced symptoms of fever, sore throat, cough, diarrhea, or fresh wound? If so, how was the problem managed? Do participants often self-medicate with antibiotics? If so, which antibiotics- name and brand? Where were they obtained, and how and what were they used for?
4. Use and cultural meaning of antibiotics.
   4.1. How do participants view and describe/explain antibiotics? What are they for? How many kinds of them? How do they differ from other types of medicines? How do antibiotics are locally named? Among each antibiotic sample shown, how are they explained in terms of efficacy and suitability to specific illness? What are the most effective ones? Why so? Is antibiotic necessary for all kinds of diseases particularly fever, sore throat, diarrhea, cough, and fresh wound? In addition to these illnesses, what else those antibiotics is good for?
5. Popular concepts of AMR, Microbes and Infection
   5.1. Do participants know or have ever heard about AMR? How do they describe it? Is it a problem? How serious and why? How does AMR relate to antibiotic use? Why? In their view, what are the causes and impact of AMR?
   5.2. What does infection mean to the participants? Are there any local traditional concepts/ terminologies close to infection? If so, what are they? If so, what are symptoms of such locally defined infection, and how are they treated?
   5.3. Do the participants have any idea about bacteria or/and microbes? If so, what are they, and how do they link with infection? Are there local/traditional terminologies having a meaning similar to bacteria or/and microbes?
