# Supplementary material for: The unseen use of antimicrobials: Drivers of human antibiotic use in a community in Thailand and implications for surveillance
Source: Glob Public Health. Author manuscript; Available in PMC 2026 Sep 7. (PMC7619281; doi:10.1080/17441692.2023.2298940)
Supplement: Supplementary file 1 [file EMS215594-supplement-Supplemental_material.zip › Interview_topic_guide.docx]

**Interview topic guide**

**Objectives:**

To gather data about the socio-economic background, daily life, illness and health-seeking and self-medication practices of the family, focused on antibiotics use, AMR knowledge and awareness and local traditional concepts of infection, food-related behaviour and water use practices.

Guideline Questions:

1. Family profiles/ background

a. How many members are in the family? Who are they in terms of name, age, gender, and education? How does each live their life? What are their sources of income? How many are farmers, factory workers, businesspeople, wage earners, unemployed? How well to do the family is?

b. What is the daily life of the family and each member? How do the whole family and each member spend their days?

2. Health

a. How healthy the family and each member are? Does anyone have health problems, currently or in the past few years? If so, who and what problems?

b. How do the family and each member do when they get sick? Where do they commonly get treatment and medicines? Where do they usually get drug for self-medication? Do they have insurance? If so, what kinds of it- universal coverage? Social security? Out-of-pocket?

c. Has anyone in the family had health problems or hospitalized in the previous three months? If so, who and what were the problems, how were the problems managed and where were they hospitalized?

d. In the last months, was anyone in the family ill from fever, sore throat, diarrhoea, cough, or fresh wound? If so, how was the problem managed? Were any kinds of antibiotics taken? If so, which antibiotics- name and brand? Where were they obtained and how and what were they used for?

3. Self-medication practice and antibiotic use, Infection and AMR,

a. How often do the family and family members self-medicate? where do they get medicines from? Why and when self-medication is a choice?

b. How do the family and family members view and perceive antibiotics? What are antibiotics for? How many kinds of them? How do they differ from other types of medicines? How do antibiotics are locally named? What are the differences among each kind/ name in terms of efficacy and suitability to specific illness? What are the most effective ones? Why so? Is antibiotic necessary for all kinds of diseases particularly fever, sore throat, diarrhoea, cough, and fresh wound? In addition to these illnesses, what else are those antibiotics needed for?

c. How does the family think when they hear about AMR? Has the term ever heard before? If so, how do the family/ members feel about it? Is it a problem? How serious it is and why? How does AMR relate to antibiotic use? Why? In their view, what are the causes and impact of AMR?

d. What does infection mean to the family/members? Are there any local traditional concepts/ terminologies close to infection? If so, what are they and how does each say? If so, what are symptoms of such locally defined infection and how are they treated?

e. Do the family/members have any idea about bacteria or/and microbes? If so, what are they and how do they link with infection? Are there local/traditional terminologies having a meaning similar to bacteria or/and microbes?

f. Has anyone in the family experienced an infection with AMR bacteria before? If so, who? When did it happen? how serious was it? and how it was treated?

4. Food and water

a. How do family/members manage their daily meals? Do they cook, dine out or both? If they cook, how often, for which meals? Does the whole family have meals together? If so, how often? If not, how each member manage themselves?

b. If cooked, where does the family get food ingredients- fresh market, superstore, local market, backyard, own farm?

c. Do the family/members usually buy cooked food from the market? If so, where and what kinds?

d. What are the main types/sources of drinking and consuming water in the household? Tapped, bottled, river?
