# Supplementary material for: The unseen use of antimicrobials: Drivers of human antibiotic use in a community in Thailand and implications for surveillance
Source: Glob Public Health. Author manuscript; Available in PMC 2026 Sep 7. (PMC7619281; doi:10.1080/17441692.2023.2298940)
Supplement: Supplementary file 1 [file EMS215594-supplement-Supplemental_material.zip › Medicine_use_survey_form.docx]

Moo _______________ Sub-district _____________ ______

Home ID Number _____________________________________

Interviewer ________________________________________

Date __________ Month __________________ Year __________

**1. General Information**

| **ID** | **Sex**  1 = Male  2 = Female  3 = Other | **Age** | **Level of education**  1 = Not in class  2 = primary school  3 = Junior High School  4 = High School  5 = Bachelor's degree  6 = Higher than bachelor degree | **Career**  1 = Farmer (rice, orchardist, etc.)  2 = Farming (shrimp, fish, duck, etc.)  3 = Government worker/State  4 = Trade  5 = Factory/Company Employee  6 = Housekeeper/Student  7 = Unemployed  8 = Wage earner  9 = Others | **Health**  1 = Healthy  2 = Not healthy but without medical conditions  3 = Have a medical condition (specify) | **Treatment rights**  1 = Gold Card  2 = Social Security  3 = Government/State Enterprise Welfare  4 = Pay yourself  5 = Others |
| --- | --- | --- | --- | --- | --- | --- |
| **1** |  |  |  |  |  |  |
| **2** |  |  |  |  |  |  |
| **3** |  |  |  |  |  |  |
| **4** |  |  |  |  |  |  |
| **5** |  |  |  |  |  |  |
| **6** |  |  |  |  |  |  |
| **7** |  |  |  |  |  |  |
| **8** |  |  |  |  |  |  |
| **9** |  |  |  |  |  |  |
| **10** |  |  |  |  |  |  |

**2. Illnesses episodes**

2.1 During the past three months, has anybody in your household had a health problem? ( )Yes	( ) No

- ( ) If chronic condition, end of survey
- ( ) If no, end of survey
- ( ) If yes, Ask them:

2.1 What symptoms did they have? Prompt: any other Symptoms? Write the answer in the box

2.3. Based on the participant’s description, please fill in the table with the symptoms they mentioned.

| **ID** | **Fever**  0 = None  1 = Yes | **Cough**  0 = None  1 = Yes | **Sore throat**  0 = None  1 = Yes | **Nasal congestion runny nose**  0 = None  1 = Yes | **Difficulty breathing**  0 = None  1 = Yes | **diarrhoea**    0 = None  1 = Yes | | **Vomiting**  0 = None  1 = Yes | **Abdominal pain**  0 = None  1 = Yes | **Wounds, abscesses, pus**  0 = None  1 = Yes | **Swelling, redness**  0 = None  1 = Yes | **Uterine pain**  0 = None  1 = Yes | **Urinary incontinence, scrubs, pain when urinating**  0 = None  1 = Yes | **Other symptoms**  0 = None  1 = Yes |
| --- | --- | --- | --- | --- | --- | --- | --- | --- | --- | --- | --- | --- | --- | --- |
| **1** |  |  |  |  |  |  |  | |  |  |  |  |  |  |
| **2** |  |  |  |  |  |  |  | |  |  |  |  |  |  |
| **3** |  |  |  |  |  |  |  | |  |  |  |  |  |  |
| **4** |  |  |  |  |  |  |  | |  |  |  |  |  |  |
| **5** |  |  |  |  |  |  |  | |  |  |  |  |  |  |

**Continue**

| **ID** | **Fever**  0 = None  1 = Yes | **Cough**  0 = None  1 = Yes | **Sore throat**  0 = None  1 = Yes | **Nasal congestion runny nose**  0 = None  1 = Yes | **Difficulty breathing**  0 = None  1 = Yes | **Diarrhoea**  0 = None  1 = Yes | **Vomiting**  0 = None  1 = Yes | **Abdominal pain**  0 = None  1 = Yes | **Wounds, abscesses, pus**  0 = None  1 = Yes | **Swelling, redness**  0 = None  1 = Yes | **Uterine pain**  0 = None  1 = Yes | **Urinary incontinence, scrubs, pain when urinating**  0 = None  1 = Yes | **Other symptoms**  0 = None  1 = Yes |
| --- | --- | --- | --- | --- | --- | --- | --- | --- | --- | --- | --- | --- | --- |
| **6** |  |  |  |  |  |  |  |  |  |  |  |  |  |
| **7** |  |  |  |  |  |  |  |  |  |  |  |  |  |
| **8** |  |  |  |  |  |  |  |  |  |  |  |  |  |
| **9** |  |  |  |  |  |  |  |  |  |  |  |  |  |
| **10** |  |  |  |  |  |  |  |  |  |  |  |  |  |

**3. About treatment behaviour**

3.1. So you mentioned in that episode of illness that you had these symptoms (e.g. fever, abdominal pain, headache, etc..) How did you treat this episode of illness? (Fill in the table accordingly)

| **ID** | **Symptomatic syndrome**  For example: participant 1 had fever and Difficulty breathing, and other symptoms (headache), fill in row one as fever, difficulty breathing and headache  Then Participant 1 had another episode of illness (urinary symptoms and abdominal pain)  Write in row two (urinary symptoms and abdominal pain) | **How to treat**  **Fill in all that apply:**  **1 = no action**  **2 = Self-healing without medication (home remedies)**  **3 = Buy medicines from grocery stores / self-eating medicine shops**  **4 = Go to sub-district health centre**  **5= district hospital.**  **6 = Go to a private clinic**  **7 = private hospital**  **8 = Other (Specify)**  For example, participant 1 for his fever and breathing difficulties and headache, first did nothing then went to the health centre then they went to a private clinic: write in the first row: 1 then 4 then 6  Then for his second episode of illness they went to the district hospital, fill in row two as 5 | **Is there an antibiotic drug?**  **0 = Unused**  **1 = Use** | **What's the name of the antibiotic?**  Write the names of all antibiotics used in treating each episode of illness.  For example, participant 1 used penicillin and amoxicillin for treating their fever and breathing difficulties, fill in first row: penicillin + amoxicillin  Then for his second episode of illness, they used Nitrofurantoin, fill in second row as Nitrofurantoin | **If antibiotics are used**  **Where did you get them from?**  **1 = Medicines stored in the house**  **2 = Grocery store**  **3 = Pharmacy**  **4 = Private Clinic / Hospital**  **5 =health centre**  **6 = Public Hospital**  **7 = Other Specify** |
| --- | --- | --- | --- | --- | --- |
| 1 |  |  |  |  |  |
|  |  |  |  |  |  |
|  |  |  |  |  |  |
| 2 |  |  |  |  |  |
|  |  |  |  |  |  |
|  |  |  |  |  |  |
| 3 |  |  |  |  |  |
|  |  |  |  |  |  |
|  |  |  |  |  |  |
| 4 |  |  |  |  |  |
|  |  |  |  |  |  |
|  |  |  |  |  |  |
| 5 |  |  |  |  |  |
|  |  |  |  |  |  |
|  |  |  |  |  |  |
| 6 |  |  |  |  |  |
|  |  |  |  |  |  |
|  |  |  |  |  |  |
| 7 |  |  |  |  |  |
|  |  |  |  |  |  |
|  |  |  |  |  |  |
| 8 |  |  |  |  |  |
|  |  |  |  |  |  |
|  |  |  |  |  |  |
| 9 |  |  |  |  |  |
|  |  |  |  |  |  |
|  |  |  |  |  |  |
| 10 |  |  |  |  |  |
|  |  |  |  |  |  |
|  |  |  |  |  |  |

**4. About the drug (antibiotics)**

| **ID** | **Name of the antibiotic taken**  For example, participant 1 took ampicillin, amoxicillin, Nitrofurantoin, write in the first row ampicillin, in the second row amoxicillin, in the third row Nitrofurantoin, etc. | **Medicinal forms used**  1 = Tablets, Capsules  2 = liquid  3 = Injection  4 = External use  5 = Other | **Medicine unit strength (can be found on the box)**  e.g., how many mg/tablets or how many mg/5ml of liquid or international unit per ampule, etc…  For example  Ampicillin injection, check the box for strength, 500mg/amp, write 500mg/amp in the first row  Amoxicillin was given as syrup, check the box for strength, 250 mg/ml, write 250 mg/ml in the second row  Nitrofurantoin was given as tablets, check the box, 50 mg/tab, write 50 mg/tab in the third row  If you don’t know leave blank | **Number of administrations per day**  **Tablets/Capsules**  ____ Tablets /Capsules /____ Times  **water pills**  ____ Spoon /____ Times  **Injections/medications/other**  ____ Times  For example, two tablets every 6 hours | **Number of days antibiotic taken** |
| --- | --- | --- | --- | --- | --- |
| 1 |  |  |  |  |  |
|  |  |  |  |  |  |
|  |  |  |  |  |  |
|  |  |  |  |  |  |
|  |  |  |  |  |  |
| 2 |  |  |  |  |  |
|  |  |  |  |  |  |
|  |  |  |  |  |  |
|  |  |  |  |  |  |
|  |  |  |  |  |  |
| 3 |  |  |  |  |  |
|  |  |  |  |  |  |
|  |  |  |  |  |  |
|  |  |  |  |  |  |
|  |  |  |  |  |  |
| 4 |  |  |  |  |  |
|  |  |  |  |  |  |
|  |  |  |  |  |  |
|  |  |  |  |  |  |
|  |  |  |  |  |  |
| 5 |  |  |  |  |  |
|  |  |  |  |  |  |
|  |  |  |  |  |  |
|  |  |  |  |  |  |
|  |  |  |  |  |  |
| 6 |  |  |  |  |  |
|  |  |  |  |  |  |
|  |  |  |  |  |  |
|  |  |  |  |  |  |
|  |  |  |  |  |  |
| 7 |  |  |  |  |  |
|  |  |  |  |  |  |
|  |  |  |  |  |  |
|  |  |  |  |  |  |
|  |  |  |  |  |  |
|  |  |  |  |  |  |
| 8 |  |  |  |  |  |
|  |  |  |  |  |  |
|  |  |  |  |  |  |
|  |  |  |  |  |  |
|  |  |  |  |  |  |
| 9 |  |  |  |  |  |
|  |  |  |  |  |  |
|  |  |  |  |  |  |
|  |  |  |  |  |  |
|  |  |  |  |  |  |
| 10 |  |  |  |  |  |
|  |  |  |  |  |  |
|  |  |  |  |  |  |
|  |  |  |  |  |  |
|  |  |  |  |  |  |
