# Supplementary material for: The unseen use of antimicrobials: Drivers of human antibiotic use in a community in Thailand and implications for surveillance
Source: Glob Public Health. Author manuscript; Available in PMC 2026 Sep 7. (PMC7619281; doi:10.1080/17441692.2023.2298940)
Supplement: Supplementary file 1 [file EMS215594-supplement-Supplemental_material.zip › Table_1._Households_demographics.docx]

**Table 1. Sociodemographic characteristics of the 40 participating households including key informants from Nakhom Pathom province.**

| ID | Members of household living currently in the house/shared land | Age categories | Reported occupations | ID | Members of household living currently in the house/shared land | Age categories | Reported occupations |
| --- | --- | --- | --- | --- | --- | --- | --- |
| Area 1: semi-rural | | | | Area 2: rural | | | |
| HH101 | 2 | 1 elder 1 adult | -Grow Lime and vegetables in their garden (farming) -Sell home-made sweets  -Factory worker | HH221 | 5 | 2 elders 3 adults | -Fish, shrimp and chicken farming -Grocery store -Gas station |
| HH102 | 3 | 2 adults 1 child | -Shrimp farming -Factory worker | HH222 | 2 | 2 elders | -Shrimp farming -Morning glory farming |
| HH103 | 12 | 2 elders 4 adults 2 children | - Baskets braiding -grow vegetables in their garden (farming) -School meals' cook -Factory worker -A seller in the floating market -Fish farming | HH223 | 3 | 2 adults 1 child | -A truck driver -Casual breading of chicks |
| HH104 | 8 | 2 elders 4 adults 2 children | - Bags/baskets braiding -A seller in the market -Electricians | HH224 | 4 | 3 elders 1 adult | -Fish and duck farming -Fruits and vegetables farming -Lotus petals peeling |
| HH105 | 13 | 1 elder 10 adults 2 children | -A freelance cook -Factory workers -Sale assistants in a fish store -A sale representative | HH225 | 5 | 2 elders 2 adults  1 child | -Run a food stall |
| HH106 | 5 | 4 adults 1 child | -Baskets braiding -Repairing clothes, cooking -Lotus farming -Delivery drivers for a factory -Fishing | HH226 | 2 | 1 elder 1 adult | -Factory worker -Makes artificial marigold garlands |
| HH107 | 11 | 1 elder 6 adults 4 children | -Factory workers -Fish farmers -A seller in the market | HH227 | 8 | 4 adults 3 children | -Fish farming -Lotus farming -Mechanic -Factory worker |
| HH108 | 7 | 2 elders 4 adults 1 child | -Factory workers -A security guard in a Temple | HH228 | 3 | 1 elder 2 adults | -A driver -Health volunteers -An office employee |
| HH109 | 5 | 2 elders 1 adult 2 children | - Baskets braiding -Sell vegetables -Fishing -A general wage earner | HH229 | 6 | 1 elder 5 adults | -Pig farming -A driver -Teachers -State employees |
| HH110 | 4 | 2 elders 1 adult 1 child | - Baskets braiding -Truck driver and worker in construction sites | HH230 | 10 | 3 elders 5 adults 2 children | - Vegetable farming -A worker in a garage -Factory workers -A teacher |
| HH111 | 4 | 1 elder 3 adults | - Baskets braiding -A security guard -Factory workers | HH231 | 5 | 3 elders 2 adult | -Rice farming -Health volunteer -Sell vegetables and fish -A teacher -A bank employee |
| HH112 | 3 | 2 elders 1 adult | -Village head  -Health volunteer -Produce and sell products from garden | HH232 | Not interviewed due to COVID-19 outbreak | | |
| HH113 | 6 | 3 elders 3 adults 1 child | -Frog farming -Fish farming -Moringa Trees farming -Grocery store -A merchant -A massage therapist -Folding boxes | HH233 | 6 | 1 elder 4 adults 1 child | -Vegetables and rice farming -A dancer |
| HH114 | 5 | 1 elder 2 adults  2 children | -A chicken farm produces for a larger farm -Fish farming -Vegetables and moringas trees farming -A seller in the market | HH234 | 3 | 3 adults | -Morning glory farming -Rice farming -A shrimp scooper -Factory worker |
| HH115 | 7 | 2 elders 3 adults 2 children | -Collect and sell rags -Wage earners -Health volunteers | HH235 | 2 | 2 elders | -Opened a grocery store -Rice and shrimp farming |
| HH116 | 6 | 3 elders 3 adults | -Fishing -Sell fish in the market | HH236 | 6 | 1 elder 3 adults 2 children | -Shrimp and rice farming |
| HH117 | 4 | 1 elder 3 adults | -Fish farming -Duck farming -An insurance company employee | HH237 | 3 | 1 elder 2 adults | -A restaurant -Rice farming -Factory worker |
| HH118 | 6 | 1 elder 4 adults 1 child | -Fish farming -Shrimp farming -Backhoe driver -Factory workers -Sell cooked food | HH238 | 4 | 4 elders | -Health volunteer -Pig farming -Fish farming -Vegetables farming |
| HH119 | 7 | 1 elder 4 adults 2 children | -Village head -Food shop -Shrimp farming -Manila tamarind trees farming -Health volunteer -Company worker | HH239 | 4 | 2 elders 2 adults | -Factory workers |
| HH120 | 8 | 2 elders 4 adults 2 children | -Rice farming -Picking vegetables -Lighting and theatre service -Health volunteer -Sellers in the market | HH240 | 13 | 1 elder 6 adults 6 children | -Food stall -Delivery driver |
