# Supplementary material for: The unseen use of antimicrobials: Drivers of human antibiotic use in a community in Thailand and implications for surveillance
Source: Glob Public Health. Author manuscript; Available in PMC 2026 Sep 7. (PMC7619281; doi:10.1080/17441692.2023.2298940)
Supplement: Supplementary file 1 [file EMS215594-supplement-Supplemental_material.zip › Table_2._Focus_groups_demographics.docx]

**Table 2. Sociodemographic characteristics of participants in focus groups conducted in rural and semi-urban communities in Nakhom Pathom province.**

| ID | Age | Gender | occupation | ID | Age | Gender | Reported occupation |
| --- | --- | --- | --- | --- | --- | --- | --- |
| Area 1: semi-urban (focus group 1) | | | | Area 2: rural (focus group 2) | | | |
| Participant 1 | 60 | Male | Farmer, village health volunteer | Participant A | 63 | Male | Postal worker, village health volunteer |
| Participant 2 | 60 | Female | Farmer | Participant B | 59 | Female | A driver, village health volunteer |
| Participant 3 | 64 | Female | Farmer, village health volunteer | Participant C | 59 | Male | Private business, village health volunteer |
| Participant 4 | 30 | Male | Employment | Participant D | 60 | Female | Seller, village health volunteer |
| Participant 5 | 35 | Male | Farmer | Participant E | 45 | Female | Employment |
| Participant 6 | 60 | Male | Farmer |  |  |  |  |
| Participant 7 | 39 | Male | Engineer |  |  |  |  |
